# **The Potential of Menstrual Blood-Derived Stem Cells in Differentiation to Epidermal Lineage: A Preliminary Report**

**Published:** 2016-01

**Authors:** Hossein Faramarzi, Davood Mehrabani, Maryam Fard, Maryam Akhavan, Sona Zare, Shabnam Bakhshalizadeh, Amir Manafi, Somaieh Kazemnejad, Reza Shirazi

**Affiliations:** 1 Department of Infectious Diseases, Larestan School of Medical Sciensce, Larestan, Iran;; 2 Stem Cell and Transgenic Technology Research Center, Shiraz University of Medical Science, Shiraz, Iran;; 3 Department of Regenerative Medicine, University of Manitoba, Winnipeg, MB, Canada;; 4 Department of Anatomical Sciences, Qazvin University of Medical Sciences, Qazvin, Iran;; 5 Skin and Stem Cell Research Center, Tehran University of Medical Sciences, Tehran, Iran;; 6 Department of Anatomical Sciences, School of Medicine, Tehran University of Medical Sciences, Tehran, Iran;; 7 Department of Medicine, Student Research Committee, Shahid Beheshti University of Medical Sciences, Tehran, Iran;; 8 Reproductive Biotechnology Research Center, Avicenna Research Institute, ACECR, Tehran, Iran;; 9 Department of Anatomical Sciences, Iran University of Medical Sciences, Tehran, Iran

**Keywords:** Menstrual blood-derived stem cells, Differentiation, Epidermal lineage

## Abstract

**BACKGROUND:**

Menstrual blood-derived stem cells (MenSCs) are a novel source of stem cells that can be easily isolated non-invasively from female volunteered donor without ethical consideration. These mesenchymal-like stem cells have high rate of proliferation and possess multi lineage differentiation potency. This study was undertaken to isolate the MenSCs and assess their potential in differentiation into epidermal lineage.

**METHODS:**

About 5-10 ml of menstrual blood (MB) was collected using sterile Diva cups inserted into vagina during menstruation from volunteered healthy fertile women aged between 22-30 years. MB was transferred into Falcon tubes containing phosphate buffered saline (PBS) without Ca2^+^ or Mg2^+^ supplemented with 2.5 µg/ml fungizone, 100 µg/mL streptomycin, 100 U/mL penicillin and 0.5 mM EDTA. Mononuclear cells were separated using Ficoll-Hypaque density gradient centrifugation and washed out in PBS. The cell pellet was suspended in DMEM-F12 medium supplemented with 10% FBS and cultured in tissue culture plates. The isolated cells were co-cultured with keratinocytes derived from the foreskin of healthy newborn male aged 2-10 months who was a candidate for circumcision for differentiation into epidermal lineage.

**RESULTS:**

The isolated MenSCs were adhered to the plate and exhibited spindle-shaped morphology. Flow cytometric analysis revealed the expression of mesenchymal markers of CD10, CD29, CD73, and CD105 and lack of hematopoietic stem cells markers. An early success in derivation of epidermal lineage from MenSCs was visible.

**CONCLUSION:**

The MenSCs are a real source to design differentiation to epidermal cells that can be used non-invasively in various dermatological lesions and diseases.

## INTRODUCTION

Stem cells as self-renewing cells proliferating without differentiation, and under defined conditions can differentiate into various cell types.^1^ The stem cells can be categorized into two major groups of embryonic stem cells (ESCs) and adult stem cells (ASCs).^2^ Embryonic stem cells are derived from inner cell mass of the blastocysts and show pluripotency characteristics by differentiation into all cells types belong to the three germinative layers (ectoderm, mesoderm, and endoderm).^3^ On the other hand, the ASCs are deposited in most adult tissues and are long-lived with restricted differentiation potency (Mehrabani et al. 2015),^4^ and lack tumorigenicity and ethical issues seen with ESCs.^5^

Friedensten et al (1968) were the ones who primarily isolated mesenchymal stem cells (MSCs) from bone marrow (BM).^6^ They were also isolated from other tissues such as adipose tissue,^7^ umbilical cord blood,^8^ endometrial tissue,^9^ and from dental pulp.^10^ MSCs have been used for regenerative purposes in patients.^11^ Multi-lineage properties of MSCs into mesodermal and ectodermal lineages were previously demonstrated for osteoblasts,^12^ neuronal-like cells,^13^ and heart muscles.^14^

Tissues such as bone marrow, adipose tissue, umbilical cord blood, placenta, dental pulp, and peripheral blood contain a pool of ASCs.^15^^,^^16^ Isolation and cultivation of these cells by standard protocols give the opportunity to use them in research and therapeutic application. The point is that the most isolation protocols are invasive and need to do surgical operation.^17^^,^^18^ Thus demands on finding an accessible source to harvest stem cells from an adult tissue through non-invasive methodology have increased. 

The newly defined adult stem cells are menstrual blood-derived stem cells (MenSCs), giving rise to hopes in clinical application of these cells. They are mesenchymal-like stem cells that can be harvested from human menstrual blood shedding of endometrium monthly.^19^^,^^20^ MenSCs have a highly proliferation and differentiation capability under specific differentiation conditions.^21^ The easy and simple way to get MenSCs without any invasive surgical intervention or hospitalization and absence of any ethical issues to isolate them are advantages of these MSCs.^22^

Molecular profile assay shows that MenSCs express some pluripotency markers including Oct-4, SSEA-4, nanog, and c-kit and also some mesenchymal stem cells specific markers such as CD9, CD29, CD44.^23^ So MenSCs are a good source of stem cells in research for differentiation into different cells and use in regenerative medicine. The differentiation of MenSCs into adipocytes, osteocytes, chondrocytes, hepatocytes, cardiomyocytes, and pancreatic cells has been previous demonstrated.^24^

They can provide a new hope in regenerative medicine for their ability in differentiation into desired cells and tissues. Therefore, MenSCs would be a valuable choice in cell-based therapies and we can consider their potential in clinical trials especially in repair of dermatological lesions. Skin regeneration and repair has become the main goal of dermatological treatments including wrinkles, photoaging, cutaneous deep wounds, and burns as they are still major concerns in dermocosmetics.^25^ So this study was conducted to isolate MenSCs and evaluate their potential in differentiation into epidermal lineage. 

## MATERIALS AND METHODS

The MenSCs were isolated from healthy fertile women aged between 22-30 years. All were volunteer donors giving a signed informed consent sheet according to ethical guideline of Avicenna Research Institute, Tehran, Iran. About 5-10 ml of menstrual blood (MB) was collected using sterile Diva cups inserted into vagina during menstruation. MB of Diva cups were then transferred into Falcon tube containing phosphate buffered saline (PBS) without Ca2^+^ or Mg2^+^ supplemented with 2.5 µg/ml fungizone, 100 µg/mL streptomycin, 100 U/mL penicillin and 0.5 mM EDTA.

Mononuclear cells were separated using Ficoll-Hypaque density gradient centrifugation and washed out in PBS. Then, the cell pellet in the tube was suspended in DMEM-F12 medium supplemented with 10% FBS and cultured in tissue culture plates. The cells were kept in 37ºC incubator with 5% CO_2 _and saturated humidity. After removal of non-adherent cells in second day of incubation, the culture of adherent cells continued until 70% confluency. 

To induce differentiation of isolated MenSCs into epidermal lineage, we co-cultured the isolated cells with keratinocytes derived from the foreskin of healthy newborn male aged 2-10 months who was a candidate for circumcision. We isolated the foreskin keratinocytes as described before.^26^

## RESULTS

**Fig. 1:**
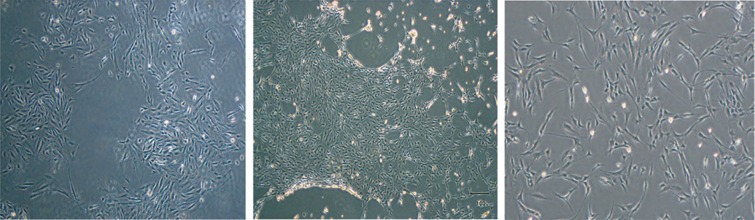
Cultured menstrual blood-derived stem cells. The adherent cells showed spindle-shaped like morphology

The isolated MenSCs were adherent to the culture plates after 24 hours and easily explanted and highly proliferated. By reaching the 75% confluency, the cells exhibited spindle-shaped morphology like fibroblasts (Figure 1). 

**Fig. 2:**
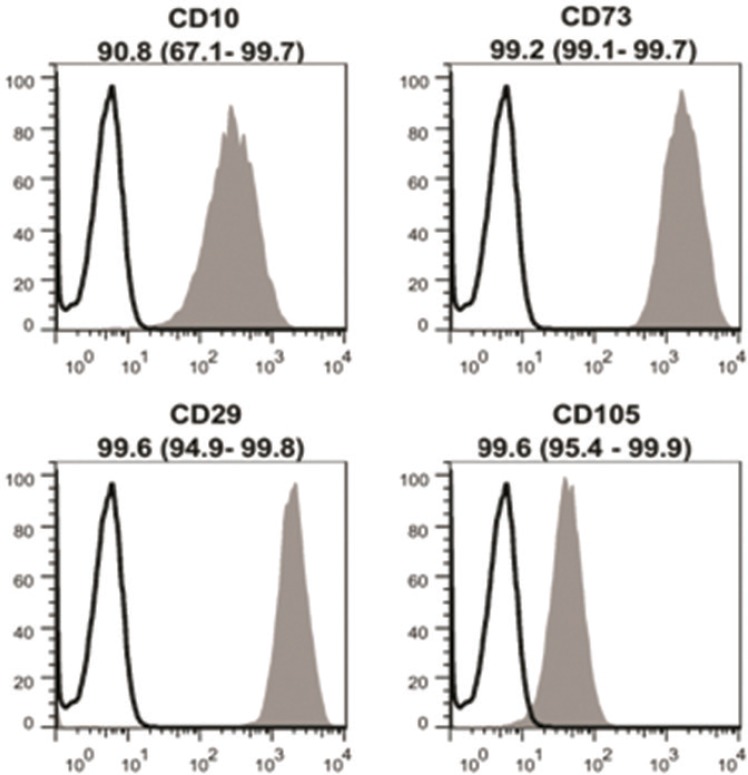
Flowcytometric analysis of isolated menstrual blood-derived stem cells. The isolated cells expressed specific markers of mesenchymal stem cells

**Fig. 3:**
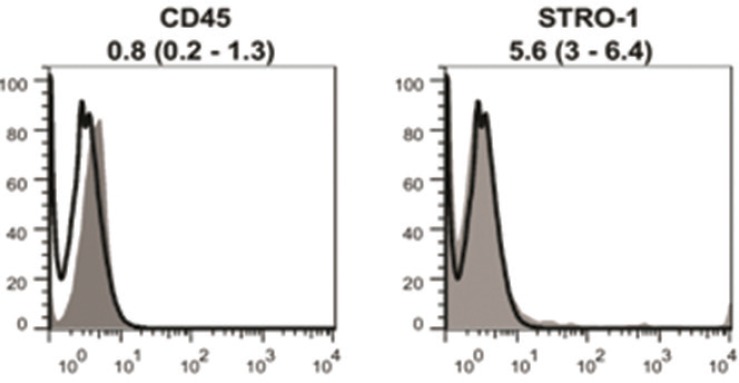
Flowcytometric analysis of isolated menstrual blood-derived stem cells. The isolated cells did not express specific markers for hematopoietic stem cells

**Fig. 4:**
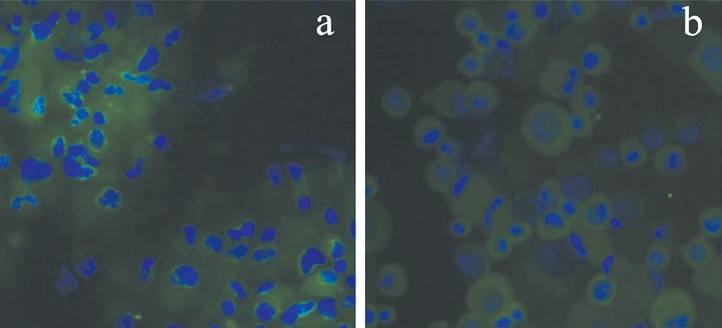
Immunostaining of epidermal markers of involucrin (a) and K14 (b

Flow cytometric analysis of cultured MenSCs revealed the expression of mesenchymal markers such as CD10, CD29, CD73, and CD105 (Figure 2). Further analysis showed the lack of hematopoietic stem cells markers (Figure 3). Under differentiation culture system, the spindle-shaped morphology of cultured MenSCs was changed into nearly irregular round to polygonal shape. At the end of the 2nd week, immunostaining assay of induced cells using indirect co-culture system revealed the expression of K14 and involucrin (Figure 4).

## DISCUSSION

The stem cells technology has opened a new window to regenerative medicine.^27^ In recent years many researchers attempted to find a safe, appropriate, and applicable way for regenerative purposes using stem cells^27^ with specific characteristics of self-renewing and differentiating ability to repair damaged tissues.^28^

Here, we designed a novel study to assess the differentiation potential of MenSCs into epidermal lineage for future repair of skin and dermatological lesions caused by ultraviolet rays, burn and chemicals damaging the integrity of skin tissue.^29^ MSCs were shown to be an effective and attractive cell population in cell therapy to induce dermal repair and regeneration following acquired lesions and wounds.^30^ They can provide essential trophic support to regenerate the injured tissue. 

Kim et al. used adipose tissue derived mesenchymal stem cells (ADSCs) to eliminate UVB-induced wrinkles. This anti-wrinkled effect was mediated by decreasing UVB-inducing apoptosis and stimulating collagen synthesis of dermal fibroblasts.^31^ Chen et al. reported the effect of cytokines and growth factors secreted by MSCs in regeneration of damaged skin tissue following full-thickness excisional wounds.^32^

The newly defined mesenchymal-like stem cells from MB called MenSCs are a new source of stem cells^33^ with good proliferation rate and capability in differentiation into various cell types similar to many other kinds of adult stem cells.^22^ Kazemnejad et al. investigated hepatic differentiation capacity of MenSCs compared to mesenchymal stem cells derived from bone marrow.^24^ The derivation of adipogenic lineage, glial cells, and cardiogenic lineage were also demonstrated in other studies.^34^^-^^36^

In addition, these easily accessible adult stem cells have the capacity to trans-differentiate into neuronal cells, pancreatic cells, and osteocytes.^33^ These investigations suggest this new source as a safe alternative to other adult stem cells for cell therapies in different diseases. So we concluded the MenSCs could provide a suitable cell sources in repair and regenerate of skin diseases and naturally photoaging of skin. We are on the line to develop and suggest the standard inducing media and protocols to derive epidermal lineage from MenSCs. By achieving this goal, novel cell-based therapies will be proposed to use in many experimental and clinical studies.

## CONFLICT OF INTEREST

The authors declare no conflict of interest.
